# Digital health technologies and Alzheimer’s disease clinical trials: might decentralized clinical trials increase participation by people with cognitive impairment?

**DOI:** 10.1186/s13195-023-01227-4

**Published:** 2023-04-27

**Authors:** Victoire Leroy, Wassim Gana, Amal Aïdoud, Jacques-Alexis N’kodo, Anna-Chloé Balageas, Pascal Blanc, Dominique Bomia, Camille Debacq, Bertrand Fougère

**Affiliations:** 1 Department of Geriatrics, Tours University Hospital, Tours, France; 2 Memory Clinic, Tours University Hospital, Tours, France; 3 EA4245 T2i, Université de Tours, Tours, France; 4 EA 7505 Education, Ethics, Health, Tours University, Tours, France

**Keywords:** Therapeutic trials, Alzheimer’s disease, Digital tools, Decentralized trials

## Abstract

**Background:**

Therapeutic trials in Alzheimer’s disease (AD) face many obstacles—particularly with regard to screening and recruitment.

**Discussion:**

Decentralized clinical trials (DCTs) are being developed in other diseases and appear to be of value for overcoming these difficulties. The use of remote visits offers hope of broader recruitment and thus a reduction in inequalities due to age, geography, and ethnicity. Furthermore, it might be easier to involve primary care providers and caregivers in DCTs. However, further studies are needed to determine the feasibility of DCTs in AD.

**Summary:**

A mixed-model DCT might constitute the first step towards completely remote trials in AD and should be assessed first.

## Background

According to Alzheimer Disease International (ADI), the number of people with dementia was 50 million in 2018 and will rise to 150 million in 2050 [1]—even though more recent data suggest that the incidence and prevalence of dementia are falling in western Europe [2]. Among people aged 60 or over, the estimated prevalence of mild cognitive impairment (which can convert to dementia) is between 16 and 20% [3]. Cognitive impairment is still challenging to prevent, diagnose, and care for, and so this major public health issue requires significant clinical research efforts.

On one hand, the global pandemic of coronavirus disease, since early 2019, exposed weaknesses in the clinical research system: ongoing trials halted their recruitment and study procedures, while new trials were left on hold [4]. On the other hand, this crisis period catalyzed the emergence of innovative clinical trials methodologies and tools [5, 6]. With the use of today’s digital health technologies (telemedicine, wearable devices, mobile apps, etc.), the decentralized clinical trial (DCT) might now be a valuable way of bypassing pinch points in clinical research [7]. In a DCT, participants do not have to visit a central investigating center (such as a hospital or research institute) on a regular basis. Study visits can be avoided though the use of digital tools for the remote collection and monitoring of the participants’ health data. DCTs can also involve decentralized trial networks that involve several investigating centers for the real-time sharing and analysis of data. Digital health technologies allow for the remote collection of information at every stage of the clinical trial and can improve recruitment and participation rates via a “patient-centric trial” approach [8].

## Discussion

In the field of cognitive disorders, the clinical research issues are huge. ADI advises investing 1% of the societal cost of dementia in clinical research [1]. Alzheimer’s disease (AD) is a major health issue because of its current high prevalence and the poor therapeutic arsenal. However, the failure rate for therapeutic trials in AD is particularly high: 99.6% in 2014, according to Cummings et al. [9]. Nevertheless, in 2022, 143 drugs were being tested in 172 trials [10]. Only two molecules have given encouraging results since then [11, 12]. Several other challenges have been identified, such as slow recruitment and missing data [13]. We believe that new communication technologies and the development of telemedicine and connected health will improve randomized clinical trials at all steps.

Recruitment is often a slow process, with an estimated rate of 0.2 patients per site per month. Due to screening failures, it has been estimated that the screening phase should be from 1.8 to 3.8 times longer than the treatment phase (depending on the trial phase and the population’s cognitive status) [10, 13]. Myers et al. [14] suggested that virtual visits can eliminate geographical barriers and enable safe, efficient recruitment in clinical trials in Parkinson’s disease. Indeed, this suggestion has been made for all clinical trials [15] and could be applied to AD trials. Moreover, recruitment into AD trials has limitations related to ethnicity and age. Indeed, a recent cross-sectional study concluded that some ethnic groups, people with a lower educational level, and women were under-represented in AD trials [16]. DCTs might constitute a good way of reducing inequalities in research participation [17]. This is the view held by some of the speakers at the 2019 “Virtual Clinical Trials: Challenges and Opportunities*”* workshop hosted by the US National Academy of Sciences [18]. Furthermore, a qualitative study in UK highlighted the need of a caregiver in recruitment [19]. DCT may also be especially beneficial for study partners and caregivers who may have difficulty traveling to a trial site due to factors such as distance, cost, or mobility issues. Therefore, one of the major benefits of a virtual trial can be precisely to reduce the burden on participants and their caregivers.

**Fig. 1.**
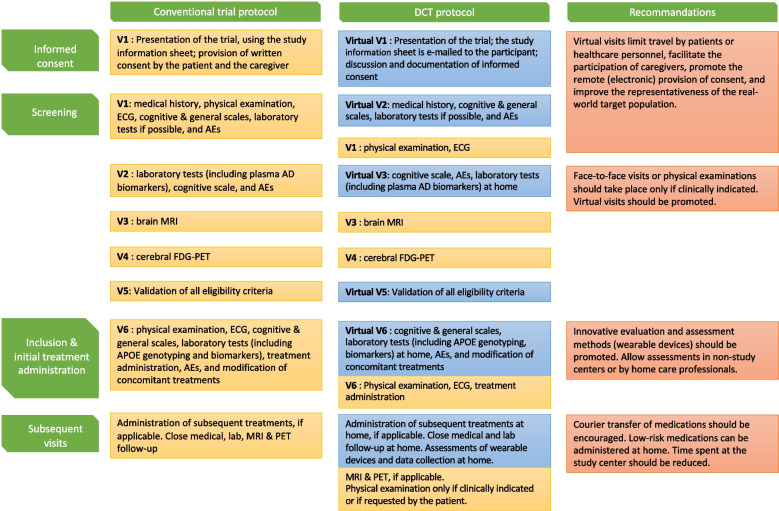
Trials designs in Alzheimer's disease. A comparison of conventional and DCT designs in AD, with recommendations for optimizing research processes. AD, Alzheimer’s disease; AE, adverse event; APOE, apolipoprotein E; ECG, electrocardiogram; FDG-PET, fluorodeoxyglucose positron emission tomography; MRI, magnetic resonance imaging

AD trials are also known for their high screening failure rate [13]. This failure necessitates a broad pre-screening process, depending on the setting and the recruitment pathway. In a study of four cohorts in Europe, the number needed to prescreen per amyloid-positive participant ranged from 6.9 to 88.5 [20]. The US National Institute on Aging has suggested a number of strategies for improving the trial recruitment of older adults with dementia. These recommendations include the development of community partnerships and the promotion of science to healthcare providers and caregivers [21]. In our opinion, remote screening and recruitment might also increase the screening rate and facilitate the involvement of primary care providers and centers (e.g., private-practice physicians, home care nurses, local hospitals, and nursing homes) in AD clinical trials. Digital health technologies (e.g., wearable devices) might also facilitate patient follow-up and safety monitoring during routine care [18]. As a result, the home administration of drugs (and especially low-risk drugs) would also become safer [7]. Through these advantages, DCTs might facilitate various aspects of patient recruitment and inclusion in the field of AD (Fig. 1). In addition to their value in the early phases of trials, digital tools might also enable greater standardization of tests and data collection and thus the composition of more uniform study subgroups.

However, DCTs have a number of limitations, especially when considering patients with cognitive impairment. Firstly, the data from a clinical examination cannot be collected remotely, and so a fully remote virtual clinical trial is probably not appropriate. On the same lines, remote cognitive assessments must be validated before they can be considered as judgment and inclusion criteria in a DCT. There are few data on this topic [22], but further dedicated studies are now warranted. This also raises questions about the administration and safety of the investigated therapies in general and parenteral treatments in particular. For example, it would be more difficult to monitor the occurrence of adverse events such as amyloid-related imaging abnormalities (which requires repeated brain MRI scans) in a virtual trial. Secondly, DCTs can represent new costs that might have not been considered previously by trial sponsors. Thirdly, and despite their theoretical utility in standardizing data collection, DCTs will lack the human factor to some extent—a factor that is important to cognitively impaired patients and their study partners and caregivers, whose role is required for key cognitive evaluation batteries. Moreover, the reliability and accuracy of remotely collected data can be questioned [23]. The implementation of virtual trials would thus require dedicated training of the healthcare personnel involved. Fourthly, more data are needed to confirm the quality and benefits of DCTs. Researchers are encouraged to publish their findings (including negative results and operational details) and thus guide the future development of DCTs [24]*.* The experiences of patients, their caregivers, and care providers must also be assessed, in order to identify potential levers for the virtual approach.

## Summary

Some of the obstacles in AD trials might be resolved by the use of digital health tools, such as remote recruitment and assessment. Some types of health data can be collected more flexibly and safety via wearable devices. Digital health technology might help to make clinical research more inclusive and more representative. By facilitating the recruitment of people from diverse settings, we can also hope to increase study sample sizes and statistical power. However, measuring the quality of digital trials remains an issue. Since virtual trials have other limitations, a hybrid approach (i.e., a combination of virtual visits at home and face-to-face visits at investigating centers or other centers) might be a good compromise. DCTs emerge as useful innovative approaches for observational and interventional trials in AD. However, further research (including feasibility studies) is needed before decentralized components can be exported to randomized, controlled trials. Lastly, the use of digital health tools in observational studies might trigger a new era of development in this field.

## Data Availability

Not applicable.
